# Prevalence of hearing impairment in neonatal encephalopathy due to hypoxia-ischemia: a systematic review and meta-analysis

**DOI:** 10.1038/s41390-024-03261-w

**Published:** 2024-05-20

**Authors:** Dinesh Pawale, Anurag Fursule, Jason Tan, Deepika Wagh, Sanjay Patole, Shripada Rao

**Affiliations:** 1 Department of Neonatology, Perth Children’s Hospital, Perth, WA Australia; 2 School of Medicine, University of Western Australia, Crawley, WA Australia; 3 Department of Neonatology, King Edwards Memorial Hospital, Perth, WA Australia

## Abstract

**Background:**

This systematic review was undertaken to estimate the overall prevalence of hearing impairment in survivors of neonatal HIE.

**Methods:**

PubMed, EMBASE, CINAHL, EMCARE and Cochrane databases, mednar (gray literature) were searched till January 2023. Randomized controlled trials and observational studies were included. The main outcome was estimation of overall prevalence of hearing impairment in survivors of HIE.

**Results:**

A total of 71studies (5821 infants assessed for hearing impairment) were included of which 56 were from high income countries (HIC) and 15 from low- or middle-income countries (LMIC). Overall prevalence rate of hearing impairment in cooled infants was 5% (95% CI: 3–6%, *n* = 4868) and 3% (95% CI: 1–6%, *n* = 953) in non-cooled HIE infants. The prevalence rate in cooled HIE infants in LMICs was 7% (95% CI: 2–15%) and in HICs was 4% (95% CI: 3–5%). The prevalence rate in non-cooled HIE infants in LMICs was 8% (95% CI: 2–17%) and HICs was 2% (95% CI: 0–4%).

**Conclusions:**

These results would be useful for counseling parents, and in acting as benchmark when comparing institutional data, and while monitoring future RCTs testing new interventions in HIE. There is a need for more data from LMICs and standardization of reporting hearing impairment.

**Impact:**

- The overall prevalence rate of hearing impairment in cooled infants with HIE was 5% (95% CI: 3–6%) and 3% (95% CI: 1–6%) in the non-cooled infants.
- The prevalence rate in cooled HIE infants in LMICs was 7% (95% CI: 2–15%) and in HICs was 4% (95% CI: 3–5%).
- The prevalence rate in non-cooled HIE infants in LMICs was 8% (95% CI: 2–17%) and HICs was 2% (95% CI: 0–4%).
- These results would be useful for counseling parents, and in acting as benchmark when comparing institutional data, and while monitoring future RCTs testing new interventions in HIE.

## Introduction

Hypoxic ischemic encephalopathy (HIE) is a leading cause of neonatal brain injury, with an incidence of 1.5 per 1000 live births in developed countries and 2.3–26.5 per 1000 live births in lower and middle-income countries.^1,2^ The sequelae of HIE encompass motor deficits, intellectual disability, and hearing and vision impairments.^3–6^ Hearing loss has the potential to hinder a child’s linguistic progress, communication abilities, social wellbeing and overall quality of life, especially in socio-demographically disadvantaged children^7–9^ Despite its significance, the overall prevalence of hearing impairment in survivors of HIE remains unclear. The 2013 Cochrane meta-analysis of seven Randomized controlled trials (RCTs) found that the incidence of hearing impairment was 3.8% (15/396) among survivors who received hypothermia and 5.8% (19/324) in those who received normothermia.^4^ The results of the Cochrane review suggested that there was no significant impact of cooling on hearing impairment (RR 0.66, 95% CI: 0.35, 1.26).^4^ The recent network meta-analysis included only seven RCTs evaluating hearing impairment, but the incidence of hearing impairment in neonatal HIE was not reported.^10^ The other recent systematic reviews did not report on hearing outcomes.^11–13^ While managing an infant with HIE, an important question that clinicians and parents face is “what are the chances of developing hearing impairment if the infant were to survive?”. To answer that question, it is essential to know the prevalence rates of hearing impairment in infants with HIE based on the current literature. Such information would be useful for counselling parents, and allocating resources for early intervention and in acting as benchmark when comparing instituitional data. Hence, this systematic review was undertaken to estimate the overall prevalence of hearing impairment in survivors of neonatal HIE.

## Methods

Guidelines from the Joanna Biggs Institute were followed for conducting and reporting this systematic review.^14^ Ethics approval was not required. The protocol was registered in PROSPERO (CRD42022335943).

### Literature search

MEDLINE through PubMed, Embase, CINAHL, Emcare and Cochrane databases were searched in January 2023. Gray literature was searched through Mednar (https://mednar.com). Two reviewers conducted the literature search independently. The reference lists of included studies and other relevant articles were searched to identify additional studies. No language restrictions were applied.

MEDLINE was searched through PubMed using the following search terms:

((((((HIE) OR (hypoxic ischemic encephalopathy)) OR (birth asphyxia)) OR (perinatal asphyxia)) OR (neonatal encephalopathy)) AND ((((((((((deafness) OR (hearing loss)) OR (hearing impairment)) OR (sensorineural deafness)) OR (sensorineural hearing loss)) OR (sensorineural hearing impairment)) OR (auditory dysfunction)) OR (auditory impairment)) OR (cochlear implant)) OR (outcome))) OR ((hypoxic ischemic encephalopathy) AND (disability)).

The automatic mapping system of PubMed expanded it to the following terms:

((“HIE” OR (“hypoxic ischemic encephalopathy” OR “hypoxia ischemia, brain” OR (“hypoxia ischemia” AND “brain”) OR “brain hypoxia-ischemia” OR (“hypoxic” AND “ischemic” AND “encephalopathy”) OR “hypoxic ischemic encephalopathy”) OR (“asphyxia neonatorum” OR (“asphyxia” AND “neonatorum”) OR “asphyxia neonatorum” OR (“birth” AND “asphyxia”) OR “birth asphyxia”) OR ((“perinatal” OR “perinatally” OR “perinatals”) AND (“asphyxia” OR “asphyxia” OR “asphyxias”)) OR ((“infant, newborn” OR (“infant” AND “newborn”) OR “newborn infant” OR “neonatal” OR “neonate” OR “neonates” OR “neonatality” OR “neonatals” OR “neonate s”) AND (“brain diseases” OR (“brain” AND “diseases”) OR “brain diseases” OR “encephalopathies” OR “encephalopathy”))) AND (“deafness” OR “deafness” OR “deafnesses” OR (“hearing loss” OR (“hearing” AND “loss”) OR “hearing loss”) OR (“hearing loss” OR (“hearing” AND “loss”) OR “hearing loss” OR (“hearing” AND “impairment”) OR “hearing impairment”) OR (“hearing loss, sensorineural” OR (“hearing” AND “loss” AND “sensorineural”) OR “sensorineural hearing loss” OR (“sensorineural” AND “deafness”) OR “sensorineural deafness”) OR (“hearing loss, sensorineural” OR (“hearing” AND “loss” AND “sensorineural”) OR “sensorineural hearing loss” OR (“sensorineural” AND “hearing” AND “loss”)) OR (“hearing loss, sensorineural” OR (“hearing” AND “loss” AND “sensorineural”) OR “sensorineural hearing loss” OR (“sensorineural” AND “hearing” AND “impairment”) OR “sensorineural hearing impairment”) OR (“hearing disorders” OR (“hearing” AND” disorders”) OR “hearing disorders” OR (“auditory” AND “dysfunction”) OR “auditory dysfunction”) OR ((“auditorially” OR “auditory”) AND (“impair” OR “impaired” OR “impairement” OR “impairements” OR “impairing” OR “impairment” OR “impairments” OR “impairs”)))) OR ((“hypoxic ischemic encephalopathy” OR “hypoxia ischemia, brain” OR (“hypoxia ischemia” AND “brain”) OR “brain hypoxia-ischemia” OR (“hypoxic” AND “ischemic” AND “encephalopathy”) OR “hypoxic ischemic encephalopathy”) AND (“disabilities” OR “disability” OR “disabled persons” OR (“disabled” AND “persons”) OR “disabled persons” OR “disabled” OR “disablement” OR “disablements” OR “disabling” OR “disability”)). Similar terms were used for other databases.

### Inclusion criteria

Eligible studies from year 2000 onwards which reported the incidence of hearing impairment in survivors of HIE were included. Studies in which neonates received hypothermia as standard of care or as part of clinical trial were included. Similarly, all studies in which neonates with HIE received normothermia as standard of care or as part of clinical trial were included. Studies published from year 2000 onwards were included to ensure appropriate comparison and contemporaneousness of the data, considering the publication of the first pilot study on therapeutic hypothermia in HIE at the time.^15^ Review articles, editorials, case reports, letters and commentaries were excluded.

The diagnosis of hearing impairment could have been based on the auditory brainstem response (ABR) or otoacoustic emissions (OAE) prior to discharge or subsequent audiology assessments or could have been reported as part of formal developmental assessments.

### Quality assessment

We used the quality assessment tool from the Joanna Briggs Institute.^16^ which has the following criteria: (1) Was the sample frame appropriate to address the target population? (2) Was the study population sampled in an appropriate way? (3) Was the sample size adequate? (4) Were the study subjects and settings described in detail? (5) Was the data analysis conducted with sufficient coverage of the identified sample? (6) Were valid methods used for identification of the condition? (7) Was the condition measured in a standard, reliable way for all the participants? (8) Was there appropriate statistical analysis? (9) Was the response rate adequate, and if not, was the low response rate managed appropriately? The criteria were rated as either yes, no, not clear, or not applicable.

### Data extraction

Two reviewers independently extracted the data using a prespecified data collection form. Information about the study design and outcomes was verified by three reviewers independently. Disagreements were resolved through discussions. Where necessary, authors of the included studies were contacted, requesting additional information from their studies.

### Data synthesis

Meta-analysis was conducted using Stata 18 (StataCorp, 4905 Lakeway Drive, College Station, Texas 77845 USA).^17^ Using the *metaprop* command to derive the pooled estimation of prevalence.^18^ The Freeman–Tukey Double Arcsine Transformation was used to stabilize the variances. The prevalence rates of hearing impairment in individual studies were computed using the formula: (number of infants with hearing impairment/number of infants assessed for hearing impairment). We used the absolute number of observed events and calculated the proportions and 95% confidence intervals (CIs), assuming a binomial distribution. A logistic normal random effects model was fitted. Heterogeneity was assessed by using the χ2 test and the I^2^ statistic. In addition, the proportions with their 95% CI values from individual studies were also presented in a forest plot. Funnel plots were used for assessing publication bias. Eggers’s test was used as formal test of funnel plot asymmetry and publication bias. Summary estimates of prevalence rates of hearing impairment were calculated separately for cooled and non-cooled neonates.

## Results

**Fig. 1:**
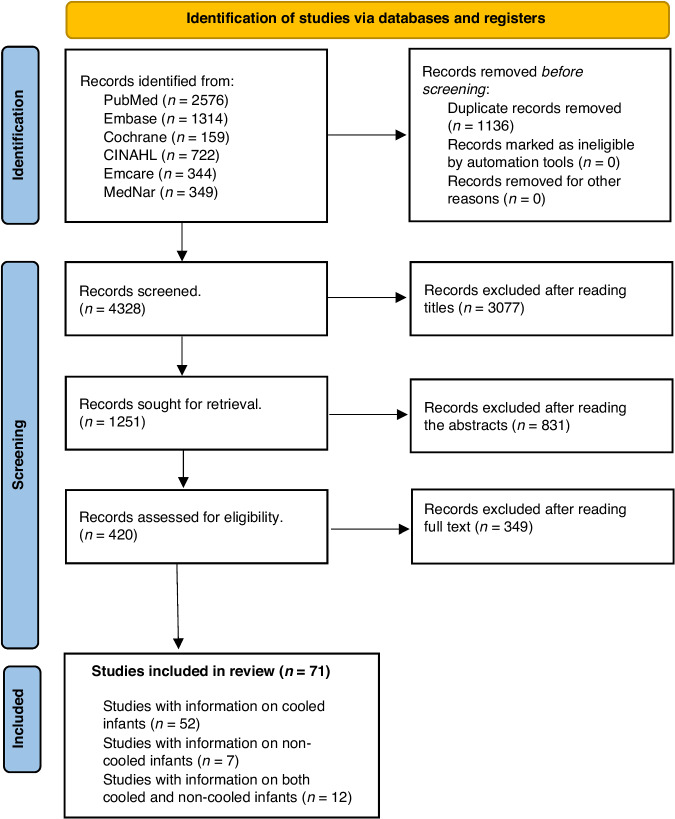
Preferred reporting items for systematic reviews and meta-analyses flow diagram.

Figure 1 provides details of the study selection process. Included studies were segregated into two groups based on whether infants received cooling or not. Infants in the non-cooled arms of RCTs were grouped together with observational studies in which infants were not cooled. Similarly, infants in the cooled arms of RCTs were grouped together with observational studies wherein infants were cooled. A total of 71 studies were included in the systematic review, of which 52 provided information on cooled infants, seven provided information on non-cooled infants and the remaining 11 provided information on both cooled and non-cooled infants in the published manuscripts. Upon request, one author provided information from their published RCT.^19^

### Studies in which infants with HIE were cooled

A total of 64 studies reported the outcomes on hearing assessment in cooled HIE infants (Table 1).^19–71^ Amongst included studies, 15 were RCTs^19,22,31,32,35,43,47,60,61,64,70,72–75^ and 49 were observational studies.^20,21,23–30,33,34,36–42,44–46,48–59,62,63,65–69,71,72,76–82^ The total sample size was 7990 cooled infants, out of which 4868 were assessed for hearing impairment. The median sample size in the included studies was 61 (IQR 27–104; range: 5–1484).

**Table 1:** Study characteristics with cooled Hypoxic Ischemic Encephalopathy infants.

Study	Study Location	Type of study	Year of birth	Income status	Type of cooling	Intervention with cooling	Age at follow-up	Diagnostic tool	Number assessed for hearing	Number Hearing impairment	Number cooled	Survival before discharge	Percentage of survivors assessed for hearing
Ancora et al.^20^	Italy	Prospective cohort study	2006–2009	High income country	Selective head cooling	None	3 months	ABR	19	1	20	19	100
Arriaga-Redondo et al.^21^	Spain	Retrospective cohort study	2009–2019	High income country	Whole body/selective head cooling	None	24 months	Not available	57	4	79	Not available	Not available
Azzopardi et al.^22^	Multicentric	Randomized controlled trial	2002–2006	High income country	Whole body	None	18 months	Not available	114	4	163	121	94.2
Barta et al.^23^	Hungary	Retrospective cohort study	2006–2010	High income country	Whole body	None	18–26 months	Not available	42	1	51	42	100
Battin et al.^72^	New Zealand	Randomized controlled trial	1996–1998	High income country	Selective head cooling	None	18 months	ABR	22	0	25	22	100
Buchiboyina et al.^24^ (A)	Australia	Retrospective cohort study	2008–2011	High income country	Whole body (MCC)	None	12–24 months	Not available	77	4	95	Not available	Not available
Buchiboyina et al.^24^ (B)	Australia	Retrospective cohort study	2011–2014	High income country	Whole body (SCC)	None	12–24 months	Not available	63	2	105	Not available	Not available
Cainelli et al.^25^	Italy	Prospective cohort study	2010–2013	High income country	Whole body	None	24 months	Not available	35	0	35	35	100
Celik et al.^76^	Turkey	Prospective cohort study	2013–2014	Upper-middle income country	Whole body	None	18–36 months	Not available	17	1	21	Not available	Not available
Chen et al.^26^	Taiwan	Retrospective cohort study	2015–2020	High income country	Not available	None	12 months	ABR	46	10	46	Not applicable	Not available
Das et al.^73^	India	Randomized controlled trial	2009–2014	Lower-middle income country	Selective head cooling	None	30 months	Not available	27	3	30	27	100
Dereymaeker et al.^27^	Belgium	Retrospective cohort study	2011–2015	High income country	Whole body	None	12–24 months	Not available	10	1	21	Not available	Not available
Dingley et al.^28^	UK	Prospective single-arm, dose-escalation feasibility study	2010–2011	High income country	Whole body	Xenon	18–20 months	Not available	11	1	14	11	100
Edmonds et al.^29^	UK	Retrospective cohort study	2009–2013	High income country	Whole body	None	5–7 years	Not available	31	3	55	Not available	Not available
Erdi-Krausz et al.^30^	UK	Retrospective cohort study	2009–2013	High income country	Whole body	None	24 months	Not available	24	2	27	Not available	Not available
Eicher et al.^31^	Multicentric	Randomized controlled trial	1998–2001	High income country	Whole body	None	12–18 months	Not available	17	2	32	22	77.3
Filippi et al.^32^ (A)	Multicentric	Randomized controlled trial	2010–2013	High income country	Whole body	None	18–24 months	Not available	22	2	23	Not available	Not available
Filippi et al.^32^ (B)	Multicentric	Randomized controlled trial	2010–2013	High income country	Whole body	Topiramate	18–24 months	Not available	18	2	21	Not available	Not available
Fitzgerald et al.^33^	Ireland	Retrospective cohort Study	2012–2016	High income country	Whole body	None	Not available	ABR/OAE	42	4	57	45	93.3
Gane et al.^74^	India	Randomized controlled trial	2011–2012	Lower-middle income country	Whole body	None	12 months	Not available	53	1	60	Feb-00	100
Giesinger et al.^34^	Canada	Prospective cohort study	2015–2016	High income country	Not available	None	18 months	Not available	31	1	46	Feb-00	91.2
Gluckman et al.^35^	Multicentric	Randomized controlled trial	1999–2002	High income country	Selective head cooling	None	18 months	Not available	64	5	112	85	75.3
Gouveia et al.^36^	Portugal	Prospective cohort study	2010–2013	High income country	Not available	None	24 months	ABR/OAE	17	0	59	50	34
Grass et al.^37^	Switzerland	Retrospective cohort study	2011–2013	High income country	Whole body	None	18–24 months	Not available	103	2	164	134	76.9
Grass et al.^38^	Canada	Retrospective cohort study	2009–2013	High income country	Whole body	None	18–36 months	Not available	142	6	182	Not available	Not available
Groenendaal et al.^39^	Netherland/ Belgium	Retrospective cohort study	2008–2011	High income country	Whole body	None	24 months	Not available	210	2	308	Not available	Not available
Grossman et al.^40^	Sweden	Prospective cohort study	2007–2009	High income country	Whole body	None	6–8 years	Not available	58	1	66	59	98.3
Guillot et al.^41^	Canada	Retrospective cohort study	2009–2016	High income country	Whole body	None	18 months	Not available	64	3	91	75	85.3
Herrera et al.^42^	USA	Retrospective cohort study	2007–2015	High income country	Whole body	None	18–24 months	Not available	17	4	30	26	65.4
Jacobs et al.^43^	Multicentric	Randomized controlled trial	2001–2007	High income country	Whole body	None	24 months	Not available	79	2	110	Not available	Not available
Koshy et al.^77^	India	Prospective cohort study	2007–2008	Lower-middle income country	Whole body	None	18–24 months	Not available	14	0	20	19	73.7
Khuwuthyakorn et al.^78^	Thailand	Retrospective cohort study	2013–2015	Upper-middle income country	Whole body	None	24 months	ABR/OAE	11	0	23	19	57.9
Kumar et al.^44^	USA	Retrospective cohort study	Not available	High income country	Not available	None	24 months	ABR	52	1	74	59	88.1
Labat et al.^45^	France	Retrospective cohort study	2017–2019	High income country	Not available	None	12 months	Not available	24	1	33	24	100
Lally et al.^46^	Multicentric	Prospective cohort study	2013–2016	High income country	Whole body	None	18–24 months	Not available	169	6	223	Not available	Not available
Laptook et al.^47^	Multicentric	Randomized controlled trial	2008–2014	High income country	Whole body	None	18–22 months	Not available	69	3	83	74	93.2
Lee et al.^48^	Taiwan	Retrospective cohort study	2015–2020	High income country	Not available	None	>12 months	ABR	74	13	74	Not available	Not available
Lee-Keeland et al.^49^	UK	Prospective case–control study	2008–2010	High income country	Whole body	None	6–8 years	Not available	29	0	40	Not available	Not available
Martinez-Hernandez et al.^79^	Mexico	Retrospective cohort study	2015–2019	Upper-middle income country	Whole body and selective head cooling	None	Not available	ABR	10	5	12	10	100
Massaro et al.^50^	USA	Retrospective cohort study	2006–2008	High income country	Whole body	None	9 months	ABR	13	3	64	Not available	Not available
Michniewicz et al.^51^	Poland	Retrospective cohort study	Not available	High income country	Whole body/selective head cooling	None	<6 months	ABR	49	4	87	Not available	Not available
Montaldo et al.^52^	Italy	Prospective cohort study	2012–2015	High income country	Whole body	None	18–24 months	Not available	64	3	73	Not available	Not available
Monzani et al.^53^	Italy	Retrospective cohort study	2012–2014	High income country	Not available	None	18–24 months	Not available	13	1	13	Not available	Not available
Natarajan et al.^54^	Multicentric USA	Retrospective cohort study	2010–2016	High income country	Whole body/selective head cooling	None	12 months	Not available	170	1	1484	1256	13.5
Peeples et al.^55^	Multicentric	Retrospective cohort study	2010–2016	High income country	Whole body/selective head cooling	None	11 months	Not available	306	6	486	Not available	Not available
Pereira et al.^56^	Portugal	Retrospective cohort study	2013–2016	High income country	Whole body	None	18–36 months	Not available	25	1	28	25	100
Perez et al.^80^	Brazil	Prospective cohort study	Not available	Upper-middle income country	Whole body (laminar flow)	None	12–24 months	Not available	18	1	26	18	100
Pokorna et al.^57^	Czech	Prospective cohort study	Not available	High income country	Whole body	None	Not available	Not available	70	6	81	70	100
Pressler et al.^58^	Multicentric	Open label dose finding feasibility trial	2011–2013	High income country	Whole body	Bumetanide	<4 weeks	ABR	11	3	14	11	100
Procianoy et al.^81^	Brazil	Prospective cohort study	2011–2017	Upper-middle income country	Whole body	None	12 months	Not available	43	5	72	53	81.1
Salam et al.^59^	USA	Retrospective cohort study	2008–2012	High income country	Whole body	None	6 months to 4 years	Not available	40	1	40	Not available	Not available
Shankaran et al.^60^	Multicentric USA	Randomized controlled trial	2000–2003	High income country	Whole body	None	18–22 months	Not available	78	3	102	78	100
Shankaran et al.^61^ -standard	Multicentric USA	Randomized controlled trial	2010–2016	High income country	Whole body	None	18–22 months	Not available	84	4	95	87	96.6
Shankaran et al.^61^ - deep cooling	Multicentric USA	Randomized controlled trial	2010–2016	High income country	Whole body	None	18–22 months	Not available	69	2	90	75	92
Shankaran et al.^61^ -prolonged	Multicentric USA	Randomized controlled trial	2010–2016	High income country	Whole body	None	18–22 months	Not available	75	4	96	78	96.2
Shankaran et al.^61^ -prolonged deep	Multicentric USA	Randomized controlled trial	2010–2016	High income country	Whole body	None	18–22 months	Not available	63	1	83	68	92.6
Shellhaas et al.^62^	USA	Retrospective cohort study	2009–2011	High income country	Whole body	None	18 months	Not available	18	0	21	Not available	Not available
Shibasaki et al.^63^	Japan	Retrospective cohort study	2012–2016	High income country	Not available	None	18–22 months	Not available	19	1	28	19	100
Simbruner et al.^64^	Germany	Randomized controlled trial	2001–2006	High income country	Whole body	None	18–21 months	Not available	30	0	62	42	71.4
Simsek et al.^82^	Turkey	Retrospective cohort study	2017–2018	Upper-middle income country	Whole body	None	Not available	ABR/OAE	126	25	126	Not available	Not available
Skrane et al.^65^	Norway	Prospective cohort study	2010–2011	High income country	Whole body	None	24 months	Not available	41	1	47	44	93.2
Smit et al.^66^	UK	Prospective cohort study	2006–1012	High income country	Whole body and selective head cooling	None	18–20 months	ABR/OAE	119	11	136	109	100
Soul et al.^19^ (A)	USA	Randomized controlled trial	2010–2017	High income country	Not available	Bumetanide	24 months	ABR	9	1	10	9	100
Soul et al.^19^ (B)	USA	Randomized controlled trial	2010–2017	High income country	Not available	None	24 months	ABR	4	0	5	4	100
Soul et al.^19^ (E)	USA	Randomized controlled trial	2010–2017	High income country	Not available	None	24 months	ABR	36	1	40	36	100
Thayyil et al.^75^	Multicentric	Randomized controlled trial	2015–2019	Lower-middle income country	Whole body	None	18–22 months	Not available	112	3	201	129	86.8
Thoresen et al.^67^	UK	Prospective cohort study	2006–2013	High income country	Whole body	None	24 months	Not available	145	7	178	161	90.1
Tsuda et al.^68^	Japan	Retrospective cohort study	2012–2016	High income country	Whole body	None	36 months	Not available	474	28	776	Not available	Not available
Valera et al.^69^	Spain	Prospective cohort study	Not available	High income country	Whole body	Erythropoietin	18 months	Not available	13	0	15	13	100
Wu et al.^70^ TH	Multicentric USA	Randomized controlled trial	2017–2019	High income country	Whole body	None	22–36 months	Not available	210	17	243	Not available	Not available
Wu et al.^70^ TH + EPO	Multicentric USA	Randomized controlled trial	2017–2019	High income country	Whole body	Erythropoietin	22–36 months	Not available	205	17	257	Not available	Not available
Xu et al.^71^	Canada	Retrospective cohort study	2004–2012	High income country	Whole body	None	Not available	Not available	132	13	181	Not available	Not available

*Buchiboyina (A)* Manually controlled cooling (MCC), *Buchiboyina (B)* Servo Controlled Cooling (SCC), *Filippi (A)* Therapeutic Hypothermia alone, *Filippi (B)* therapeutic hypothermia plus topiramate, *EPO* Erythropoietin, *TH* Therapeutic Hypothermia, *Soul (A)* Infants with HIE in bumetanide group who underwent cooling, *Soul (B)* Infants with HIE in placebo group who underwent cooling, *Soul (E)* Infants with HIE who were not randomized to treatment/placebo, who received cooling, *OAE* Otoacoustic emissions, *ABR* auditory brainstem response.

Full information on the total number of survivors from all included studies was not available. However, based on the available data from 38 studies where such information was available, the median follow up rates among survivors in individual studies were 96.3% (IQR: 86.3–100%, range: 13.5–100%).The median age at reported follow up was 22 months (IQR: 18–24 months). In the majority of studies, hearing impairment was reported as a part of developmental assessment using validated tools such as Bayley or Griffiths Scales. Fifteen studies reported about the diagnostic tools used for hearing impairment assessment, with ABR or OAE being the commonly used tools.^19,20,26,33,36,44,48,50,51,58,66,72,78,79,82^ Five studies reported about diagnosis of hearing impairment before initial discharge from hospital.^19,26,48,66,79^

### Studies in which infants with HIE were not cooled

A total of 19 studies reported the outcome of hearing impairment in non-cooled HIE infants (Table 2).^19,22,31,35,43,47,60,64,72–75,83–89^ Amongst included studies, 12 were non-cooled arms of RCTs.^19,22,31,35,43,47,60,64,72–75^ and seven were observational studies.^83–89^ The total sample size was 1402 infants, out of which 953 were assessed for hearing impairment. The median sample size in the included studies was 61 (IQR 22–110; range: 3–206). Full information on the total number of survivors from all included studies was not available. Based on 11 studies on non-cooled infants in which such information was available, median follow-up rates amongst survivors in individual studies was 100% (IQR: 83.3–100%, Range: 29.3–100%). In majority of studies, hearing impairment was reported as a part of developmental assessment. The median age at reported follow up was 20 months (IQR: 13–22 months). Three studies reported information about the diagnostic tools used for hearing impairment assessment, all of them using the ABR.^19,72,84^.

**Table 2:** Study characteristics with non-cooled hypoxic ischemic encephalopathy infants.

Study	Study Location	Type of study	Year of birth	Income status	Age at follow-up	Diagnostic tool	Number assessed for hearing	Number Hearing impairment	Number of Infants with HIE	Survival before discharge	Percentage of survivors assessed for hearing
Azzopardi et al.^22^	Multicentric	Randomized controlled trial	2002–2006	High income country	18 months	Not available	108	7	162	Not available	Not Available
Battin et al.^72^	New Zealand	Randomized controlled trial	1996–1998	High income country	18 months	ABR	12	0	15	12	100
Chalak et al.^83^	Multicentric	Prospective cohort study	2012–2015	High income country	18–22 months	Not available	51	0	63	Not available	Not Available
Das et al.^73^	India	Randomized controlled trial	2009–2014	Lower-middle income country	30 months	Not available	21	8	30	21	100
Eicher et al.^31^	Multicentric	Randomized controlled trial	1998–2001	High income country	12–18 months	Not available	11	3	33	19	57.8
Gane et al.^74^	India	Randomized controlled trial	2011–2012	Lower-middle income country	12 months	Not available	50	4	60	52	96.1
Gluckman et al.^35^	Multicentric	Randomized controlled trial	1999–2002	High income country	18 months	Not available	55	3	112	68	80.8
Jacobs et al.^43^	Multicentric	Randomized controlled trial	2001–2007	High income country	24 months	Not available	58	2	111	101	57.4
Jiang et al.^84^	China	Prospective cohort study	Not available	Upper middle-income country	1 month	ABR	46	4	46	Not available	Not Available
Jyoti et al.^85^	Australia	Prospective cohort study	1999–2001	High income country	12 months	Not available	19	0	20	Not available	Not Available
Kodama et al.^86^	Japan	retrospective cohort study	1998–2003	High income country	12 months	Not available	15	0	Not available	Not available	Not Available
Laptook et al.^47^	Multicentric	Randomized controlled trial	2008–2014	High income country	18–22 months	Not available	70	4	85	77	90.9
Martinez-Biarge et al.^87^	UK	retrospective cohort study	1992–2007	High income country	24 months	Not available	84	2	100	Not available	Not Available
Mwakyusa et al.^88^	Tanzania	Prospective cohort study	Not available	Low-income country	6 months	Not available	82	1	140	Not available	Not Available
Parmentier et al.^89^	Netherlands	retrospective cohort study	2008–2019	High income country	18–24 months	Not available	39	1	39	Not available	Not Available
Shankaran et al.^60^	Multicentric USA	Randomized controlled trial	2000–2003	High income country	18–22 months	Not available	68	4	106	68	100
Simbruner et al.^64^	Germany	Randomized controlled trial	2001–2006	High income country	18–21 months	Not available	17	2	63	58	29.3
Soul et al.^19^ (C)	USA	Randomized controlled trial	2010–2017	High income country	24 months	ABR	4	1	4	4	100
Soul et al.^19^ (D)	USA	Randomized controlled trial	2010–2017	High income country	24 months	ABR	4	0	4	4	100
Soul et al.^19^ (F)	USA	Randomized controlled trial	2010–2017	High income country	24 months	ABR	3	0	3	3	100
Thayyil et al.^75^	Multicentric	Randomized controlled trial	2015–2019	Lower-middle income country	18–22 months	Not available	136	6	206	136	100

*ABR* Auditory brainstem reflex, *Soul (C)* Infants with HIE in the bumetanide group who did not have cooling, *Soul (D)* Infants with HIE in the placebo group who did not have cooling, *Soul (F)* Infants with HIE who were not randomized to treatment/placebo, who do not received cooling.

### Meta-analysis

**Fig. 2:**
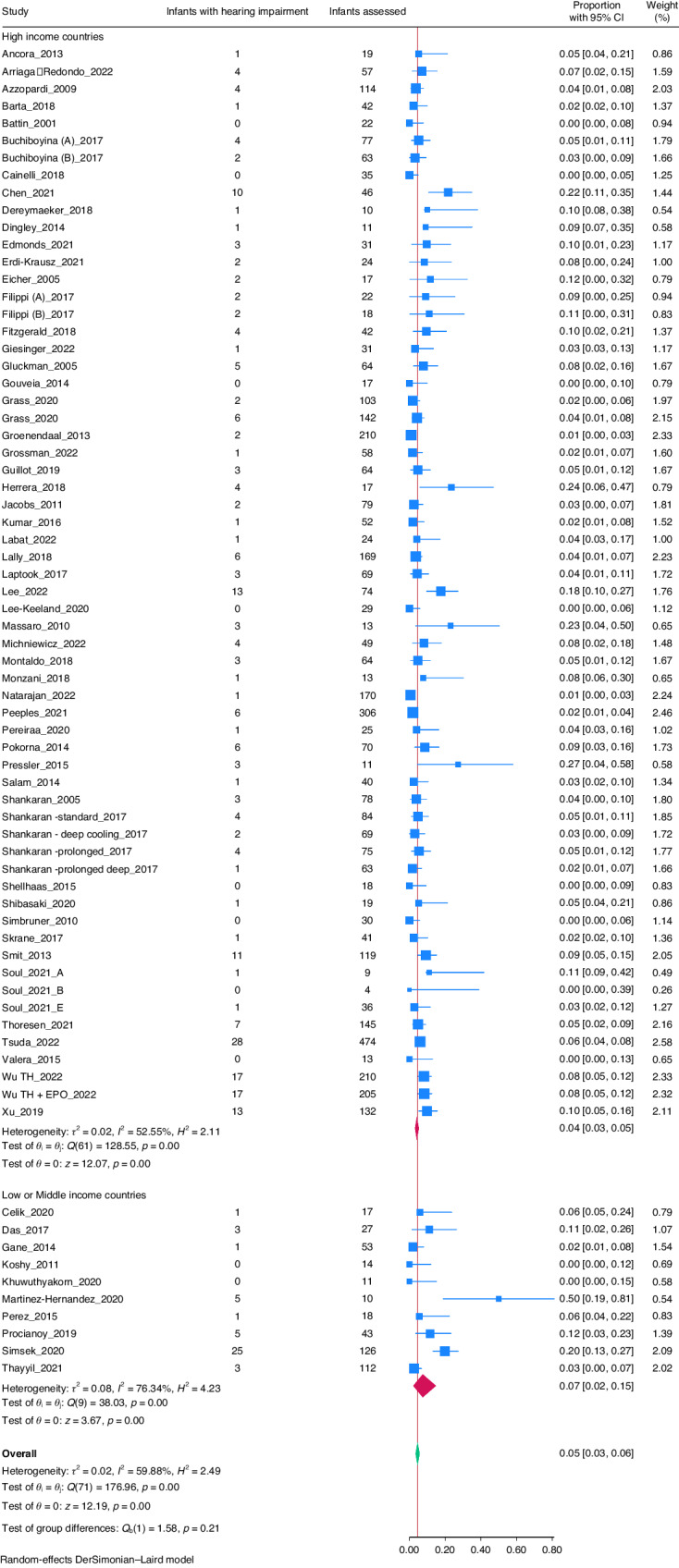
Forrest plot of studies with cooled HIE infants.

**Fig. 3:**
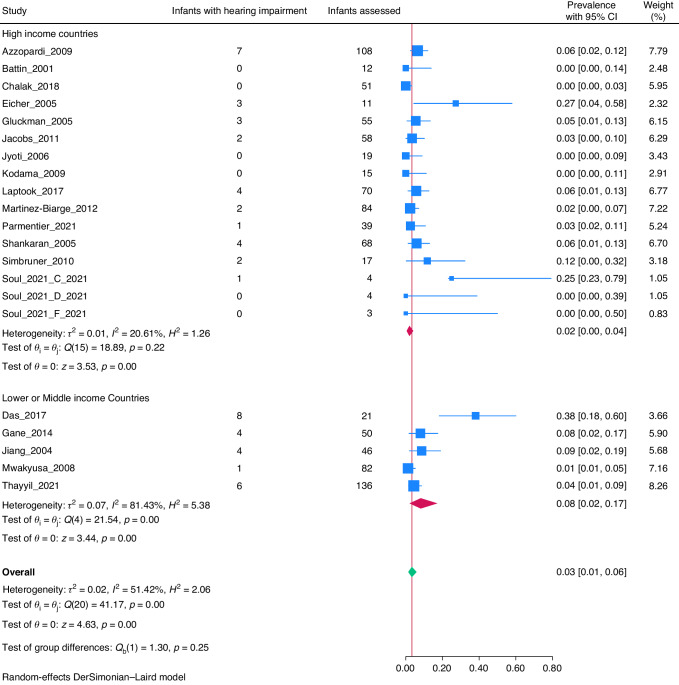
Forrest plot of studies with non-cooled HIE infants.

The overall prevalence rate of hearing impairment among survivors of cooled HIE infants was 5% (95% CI: 3–6%; I^2^ = 59.88%, Fig. 2). The prevalence rate of hearing impairment in survivors of non-cooled HIE infants was 3% (95% CI: 1–6%; I^2^ = 51.42%, Fig. 3).

### Subgroup analysis

To explore heterogeneity, a subgroup analysis was conducted based on income level of countries.^90^ Out of 71 included studies, 15 (21.1%) were conducted in low or middle-income countries (LMICs),^73–82,84,88^ whereas 56 (78.8%) were from high-income countries (HICs).^19–39,41–72,83,85–87,89^ The prevalence rate of hearing impairment in cooled infants in LMICs was 7% (95% CI: 2–15%) and in HICs was 4% (95% CI: 3–5%) (Fig. 2). The prevalence rate in non-cooled infants in LMICs was 8% (95% CI: 2–17%) and in HICs was 2% (95% CI: 0–4%) (Fig. 3).

### Publication bias

**Fig. 4:**
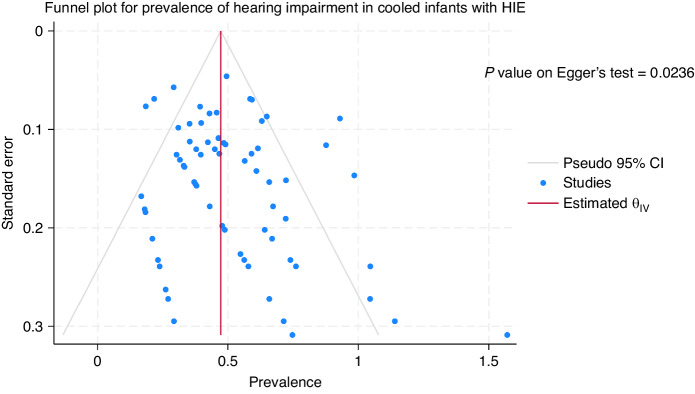
Funnel plot of studies with cooled HIE infants.

**Fig. 5:**
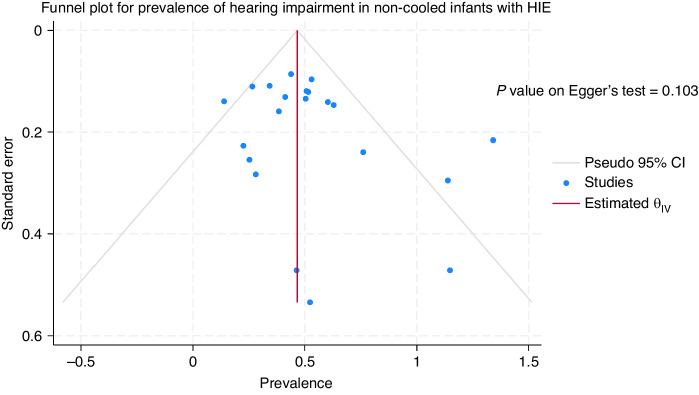
Funnel plot of studies with non-cooled HIE infants.

The funnel plots and the p values on the Egger’s test for the studies with cooled infants (*P* = 0.023) suggested publication bias while funnel plot for the studies with non-cooled infants (*P* = 0.103) did not suggest statistically significant publication bias (Figs. 4 and 5).

#### Sensitivity analysis

In further explore heterogeneity, the following analyses were conducted.

- Prevalence of hearing impairment was estimated separately for selective head cooling, whole body cooling and mixed cooling. The results were similar with a prevalence between 4 and 6%. (Table 3).

**Table 3:** Sensitivity analysis.

As per severity of HIE in cooled HIE infants			
	Number of studies	Pooled Prevalence (95% C.I.)	I^2^
Moderate to Severe HIE	52	5% (95% C.I. 4–7%)	58.17%
Any stage HIE	20	3% (95% C.I. 1–4%)	54.89%
**As per method of cooling in cooled HIE infants**			
Selective Head Cooling	4	6% (95% C.I. 2–11%)	14.4%
Whole body cooling	51	4% (95% C.I. 3–6%)	51.7%
Mixed Cooling	6	6% (95% C.I. 1–13%)	85.99%
**As per severity of HIE in non-cooled HIE infants**			
Moderate to Severe HIE	10	6% (95% C.I. 3–10%)	53.98%
Any stage HIE	9	9% (95% C.I. 0–3.8%)	39.82%
**As per percentage of follow up in cooled HIE infants**			
≥80% follow up	29	6% (95% C.I. 4–8%)	58.28%
<80% follow up	35	4% (95% C.I. 3–5%)	53.30%
**As per percentage of follow up in non-cooled HIE infants**			
≥80% follow up	10	1% (95% C.I. 0–3%)	13.38%
<80% follow up	9	7% (95% C.I. 3–12%)	70.03%
**Based on diagnostic tool used (ABR/OAE) in cooled HIE infants**			
ABR/OAE	15	9% (95% C.I. 5–14%)	66.5%
Not reported	49	4% (95% C.I. 3–5%)	38.68%
**Based on diagnostic tool used (ABR/OAE) in non-cooled HIE infants**			
ABR/OAE	3	3% (95% C.I. 0–10%)	0.00%
Not reported	16	4% (95% C.I. 2–7%)	59.84%
- In many studies, cooling was initiated even in infants with Stage I HIE.^21,31,37,38,40,43,46,48,54,55,65,68,72,76,83,84,87^ The inclusion of such infants in the meta-analysis could have resulted in an underestimation of the overall prevalence rates in moderate to severe HIE. Hence, we conducted a sensitivity analysis by analyzing them separately. The overall prevalence was 5% in moderate to severe HIE whereas it was 3% in studies that had included infants even with mild HIE (Table 3).
- The prevalence of hearing impairment in cooled HIE surviving infants among studies that had ≥80% follow-up was 6% and in <80% follow-up was 4%. The prevalence of hearing impairment in non-cooled HIE infants with studies that had more than ≥80% follow-up was 1% and that in <80% follow-up was 7% (Table 3).
- The prevalence of hearing impairment in cooled HIE surviving infants among studies that had mentioned the use of BERA/OAE was 9%. The prevalence of hearing impairment was 4% where studies reported it as part of formal developmental assessment. The prevalence in non-cooled HIE surviving infants among studies that had mentioned the use of BERA/OAE was 3%. The prevalence of hearing impairment was 4% where studies reported it as part of formal developmental assessment (Table 3).

### Risk of bias

The risk of bias was low in the majority of the domains in the included studies (Tables 4 and 5).

**Table 4:** Risk of bias assessment of studies with cooled Hypoxic Ischemic Encephalopathy infants.

Study	Was the sample frame appropriate to address the target population?	Were study participants sampled in an appropriate way?	Was the sample size adequate?	Were the study subjects and the setting described in detail?	Was the data analysis conducted with sufficient coverage of the identified sample?	Were valid methods used for the identification of the condition?	Was the condition measured in a standard, reliable way for all participants?	Was there appropriate statistical analysis?	Was the response rate adequate, and if not, was the low response rate managed appropriately?
Ancora et al. ^20^	Yes	Yes	No	Yes	Yes	Yes	Yes	Yes	Yes
Arriaga-Redondo et al.^21^	Yes	Yes	Unclear	Yes	Yes	Yes	Yes	Yes	Unclear
Azzopardi et al.^22^	Yes	Yes	Yes	Yes	Yes	Yes	Yes	Yes	Yes
Barta et al.^23^	Yes	Yes	Unclear	Yes	Yes	Yes	Yes	Yes	Yes
Battin et al.^72^	Yes	Yes	No	Yes	Yes	Yes	Yes	Yes	Yes
Buchiboyina et al.^24^	Yes	Yes	Yes	Yes	Yes	Yes	Yes	Yes	Unclear
Cainelli et al.^25^	Yes	Yes	No	Yes	Yes	Yes	Yes	Yes	Yes
Celik 2020	Yes	Yes	No	Yes	Yes	Yes	Yes	Yes	Unclear
Chen et al.^26^	Yes	Yes	Unclear	Yes	Yes	Yes	Yes	Yes	Unclear
Das et al.^73^	Yes	Yes	No	Yes	Yes	Yes	Yes	Yes	Yes
Dereymaeker et al.^27^	Yes	Yes	No	Yes	Yes	Yes	Yes	Yes	Unclear
Dingley et al.^28^	Yes	Yes	No	Yes	Yes	Yes	Yes	Yes	Yes
Edmonds et al.^29^	Yes	Yes	Unclear	Yes	Yes	Yes	Yes	Yes	Unclear
Erdi-Krausz et al.^30^	Yes	Yes	No	Yes	Yes	Yes	Yes	Yes	Unclear
Eicher et al.^31^	Yes	Yes	No	Yes	Yes	Yes	Yes	Yes	No
Filippi et al.^32^	Yes	Yes	No	Yes	Yes	Yes	Yes	Yes	Unclear
Fitzgerald et al.^33^	Yes	Yes	No	Yes	Yes	Yes	Yes	Yes	Yes
Gane et al.^74^	Yes	Yes	Yes	Yes	Yes	Yes	Yes	Yes	Yes
Giesinger et al.^34^	Yes	Yes	Unclear	Yes	Yes	Yes	Yes	Yes	Yes
Gluckman et al.^35^	Yes	Yes	Yes	Yes	Yes	Yes	Yes	Yes	No
Gouveia et al.^36^	Yes	Yes	Unclear	Unclear	Yes	Yes	Yes	Yes	No
Grass et al.^37^	Yes	Yes	Yes	Yes	Yes	Yes	Yes	Yes	No
Grass et al.^38^	Yes	Yes	Yes	Yes	Yes	Yes	Yes	Yes	Unclear
Groenendaal et al.^39^	Yes	Yes	Yes	Yes	Yes	Yes	Yes	Yes	Unclear
Grossman et al.^40^	Yes	Yes	No	Yes	Yes	Yes	Yes	Yes	Yes
Guillot et al.^41^	Yes	Yes	No	Yes	Yes	Yes	Yes	Yes	Yes
Herrera et al.^42^	Yes	Yes	No	Yes	Yes	Yes	Yes	Yes	No
Jacobs et al.^43^	Yes	Yes	Yes	Yes	Yes	Yes	Yes	Yes	Unclear
Koshy et al.^77^	Yes	Yes	Yes	Yes	Yes	Yes	Yes	Yes	No
Khuwuthyakorn et al.^78^	Yes	Yes	No	Yes	Yes	Yes	Yes	Yes	No
Kumar et al.^44^	Yes	Yes	Unclear	Yes	Yes	Yes	Yes	Yes	Yes
Labat et al.^45^	Yes	Yes	No	Yes	Yes	Yes	Yes	Yes	Yes
Lally et al.^46^	Yes	Yes	Yes	Yes	Yes	Yes	Yes	Yes	Unclear
Laptook et al.^47^	Yes	Yes	Yes	Yes	Yes	Yes	Yes	Yes	Yes
Lee et al.^48^	Yes	Yes	Unclear	Yes	Yes	Yes	Yes	Yes	Unclear
Lee-Keeland et al.^49^	Yes	Yes	No	Yes	Yes	Yes	Yes	Yes	Unclear
Massaro et al.^50^	Yes	Yes	Unclear	Yes	Yes	Yes	Yes	Yes	Unclear
Martinez-Hernandez et al.^79^	Yes	Yes	No	Yes	Yes	Yes	Yes	Yes	Yes
Michniewicz et al.^51^	Yes	Yes	Unclear	Yes	Yes	Yes	Yes	Yes	Unclear
Montaldo et al.^52^	Yes	Yes	Unclear	Yes	Yes	Yes	Yes	Yes	Unclear
Monzani et al.^53^	Yes	Yes	No	Yes	Yes	Yes	Yes	Yes	Unclear
Natarajan et al.^54^	Yes	Yes	Yes	Yes	Yes	Yes	Yes	Yes	No
Peeples et al.^55^	Yes	Yes	Yes	Yes	Yes	Yes	Yes	Yes	Unclear
Pereira et al.^56^	Yes	Yes	No	Yes	Yes	Yes	Yes	Yes	Yes
Perez et al.^80^	Yes	Yes	No	Yes	Yes	Yes	Yes	Yes	Yes
Pokorna et al.^57^	Yes	Yes	Unclear	Yes	Yes	Yes	Yes	Yes	Yes
Pressler et al.^58^	Yes	Yes	No	Yes	Yes	Yes	Yes	Yes	Yes
Procianoy et al.^81^	Yes	Yes	Unclear	Yes	Yes	Yes	Yes	Yes	Yes
Salam et al.^59^	Yes	Yes	No	Yes	Yes	Yes	Yes	Yes	Unclear
Shankaran et al.^60^	Yes	Yes	Yes	Yes	Yes	Yes	Yes	Yes	Yes
Shankaran et al.^61^	Yes	Yes	Yes	Yes	Yes	Yes	Yes	Yes	Yes
Shellhaas et al.^62^	Yes	Yes	No	Yes	Yes	Yes	Yes	Yes	Unclear
Shibasaki et al.^63^	Yes	Yes	No	Yes	Yes	Yes	Yes	Yes	Yes
Simbruner et al.^64^	Yes	Yes	Yes	Yes	Yes	Yes	Yes	Yes	Unclear
Simsek et al.^82^	Yes	Yes	Unclear	Yes	Yes	Yes	Yes	Yes	No
Skrane et al.^65^	Yes	Yes	No	Yes	Yes	Yes	Yes	Yes	Yes
Smit et al.^66^	Yes	Yes	Unclear	Yes	Yes	Yes	Yes	Yes	Yes
Soul et al.^19^	No	Yes	No	No	Yes	Yes	Yes	Yes	Yes
Thayyil et al.^75^	Yes	Yes	Yes	Yes	Yes	Yes	Yes	Yes	Yes
Thoresen et al.^67^	Yes	Yes	Unclear	Yes	Yes	Yes	Yes	Yes	Yes
Tsuda et al.^68^	Yes	Yes	Unclear	Yes	Yes	Yes	Yes	Yes	Unclear
Valera et al.^69^	Yes	Yes	No	Yes	Yes	Yes	Yes	Yes	Yes
Wu et al.^70^	Yes	Yes	Yes	Yes	Yes	Yes	Yes	Yes	Unclear
Xu et al.^71^	Yes	Yes	Unclear	Yes	Yes	Yes	Yes	Yes	Unclear

**Table 5:** Risk of bias assessment of studies with non-cooled hypoxic ischemic encephalopathy infants.

Study	Was the sample frame appropriate to address the target population?	Were study participants sampled in an appropriate way?	Was the sample size adequate?	Were the study subjects and the setting described in detail?	Was the data analysis conducted with sufficient coverage of the identified sample?	Were valid methods used for the identification of the condition?	Was the condition measured in a standard, reliable way for all participants?	Was there appropriate statistical analysis?	Was the response rate adequate, and if not, was the low response rate managed appropriately?
Azzopardi et al.^22^	Yes	Yes	Yes	Yes	Yes	Yes	Yes	Yes	Unclear
Battin et al.^72^	Yes	Yes	No	Yes	Yes	Yes	Yes	Yes	Yes
Chalak et al.^83^	Yes	Yes	Unclear	Yes	Yes	Yes	Yes	Yes	Unclear
Das et al.^73^	Yes	Yes	No	Yes	Yes	Yes	Yes	Yes	Yes
Eicher et al.^31^	Yes	Yes	No	Yes	Yes	Yes	Yes	Yes	No
Gane et al.^74^	Yes	Yes	Yes	Yes	Yes	Yes	Yes	Yes	Yes
Gluckman et al.^35^	Yes	Yes	Yes	Yes	Yes	Yes	Yes	Yes	Yes
Jacobs et al.^43^	Yes	Yes	Yes	Yes	Yes	Yes	Yes	Yes	No
Jiang et al.^84^	Yes	Yes	Unclear	Yes	Yes	Yes	Yes	Yes	Unclear
Jyoti et al.^85^	Yes	Yes	No	Yes	Yes	Yes	Yes	Yes	Unclear
Kodama et al.^86^	Yes	Yes	Unclear	Yes	Yes	Unclear	Unclear	Yes	Unclear
Laptook et al.^47^	Yes	Yes	Yes	Yes	Yes	Yes	Yes	Yes	Yes
Martinez-Biarge et al.^87^	Yes	Yes	Unclear	Yes	Yes	Yes	Yes	Yes	Unclear
Mwakusa et al.^88^	Yes	Yes	Yes	Yes	Yes	Yes	Yes	Yes	Unclear
Parmentier et al.^89^	Yes	Yes	Unclear	Yes	Yes	Yes	Yes	Yes	Unclear
Shankaran et al.^60^	Yes	Yes	Yes	Yes	Yes	Yes	Yes	Yes	Yes
Simbruner et al.^64^	Yes	Yes	Yes	Yes	Yes	Yes	Yes	Yes	No
Soul et al.^19^	No	Yes	No	No	Yes	Yes	Yes	Yes	Yes
Thayyil et al.^75^	Yes	Yes	Yes	Yes	Yes	Yes	Yes	Yes	Yes

## Discussion

In this systematic review that included 5821 infants from 71 studies, the overall prevalence rate of hearing impairment in surviving cooled infants with HIE was 5% [(95% CI 3–6%), *n* = 4868 from 64 studies)] and 3% [(95% CI 1–6%), *n* = 953 infants from 19 studies] in non-cooled infants during the same time period. These rates are significantly higher than the global prevalence of 0.1–0.4% (1–4 per 1000 live births) in general neonatal population^91^ and 1.57% (15.7 per 1000) in NICU population.^92^ These results would be useful as a benchmark for comparing institutional data, counseling parents and guiding policy makers.^93^ The results will also be useful for data and safety monitoring committees (DSMCs) of randomized controlled trials evaluating  potentially ototoxic drugs in neonates with HIE.^94^ The dose finding study of bumetanide for neonatal seizures (NEMO trial) was stopped prematurely by the DSMC due insufficient efficacy and a potential increased prevalence of hearing loss (3/11 or 27%) in infants with HIE who had received bumetanide.^58^ In that context, the results of our meta-analysis have the potential to guide the DSMCs while monitoring new interventions in neonates with HIE.

Our overall results are similar to the Cochrane meta-analysis of seven RCTs that found the incidence to be 3.8% (15/396) among survivors who had received hypothermia and 5.8% (19/324) in those who received normothermia.^4^ Even though our study was not about comparing cooling versus normothermia, the prevalence rates of 5% [(95% CI 3–6%), and 3% [(95% CI 1–6%) respectively with overlapping confidence intervals suggests that cooling may not have an impact on hearing impairment, similar to the findings of the Cochrane review.

Our meta-analysis found a higher prevalence of hearing impairment in LMICs compared to HICs. The probable reasons for this finding include the use of ototoxic antibiotics without optimal monitoring of drug levels, and higher incidence of sepsis, low birthweight and growth restricted infants in LMICs. Even though the burden of HIE is more in LMICs, there are very limited number of studies from such countries reporting on hearing impairment. Institutions managing neonates with HIE should be provided adequate resources to enable audiology and long-term developmental assessments.

This review highlights the inconsistency among studies in the reporting various aspects of hearing impairment. In the majority of studies, screening or diagnostic tools used for hearing impairment and timing of assessment were not mentioned. In many studies, the severity of hearing impairment was categorized as not requiring amplification, corrected by amplification, or not corrected by amplification.^21,22,24,32,34,35,37,42,43,45–47,52,56,60,61,64,72,73,75,80,83^ However, in some studies, the severity was more objectively classified based on the level of hearing threshold in decibels.^26,32,35,48,50,51,58,84^ Details regarding the laterality of hearing impairment were found in only 10 studies,^26,33,35,50,51,58,65,76,79,84^ while the severity was addressed in only 15 studies.^20,26,28,37,39,40,42,43,51,56,58,60,64,67,72,89^ Even among the subset of fifteen studies, only three provided a grading system for hearing impairment severity.^26,51,60^ Given the importance of hearing in acquiring language and communication skills, future studies should incorporate crucial information such as the age at screening, age at definitive diagnosis, tools used for screening and definitive diagnosis, severity, laterality, age at intervention, and type of intervention to enable comprehensive understanding. A standardized approach would facilitate more accurate comparisons across studies and enable healthcare providers to develop evidence-based strategies for the prevention and management of hearing impairment in this population.

The limitations of the systematic review include the presence of heterogeneity, insufficient information on the severity and laterality of hearing impairment and on the methods used for assessing hearing. The strengths of our review include the robust and comprehensive literature search, large sample size, inclusion of RCTs as well as real life data, formal assessment of publication bias and exploration of heterogeneity through sensitivity and subgroup analyses.

## Conclusion

The overall prevalence rate of hearing impairment in cooled surviving infants was 5% (95% CI 3–6%), and 3% (95% CI 1–6%) in the non-cooled surviving infants with HIE. These results would be useful for counseling parents, and in acting as benchmark when comparing institutional data, and while monitoring future RCTs testing new interventions in HIE.

## Supplementary information

PRISMA_2020_checklist
